# Understanding the Experience of Geriatric Care Professionals in Using Telemedicine to Care for Older Patients in Response to the COVID-19 Pandemic: Mixed Methods Study

**DOI:** 10.2196/34952

**Published:** 2022-08-10

**Authors:** Wenwen Chen, Ashley Flanagan, Pria MD Nippak, Michael Nicin, Samir K Sinha

**Affiliations:** 1 School of Health Services Management Ted Rogers School of Management Toronto Metropolitan University Toronto, ON Canada; 2 National Institute on Ageing Ted Rogers School of Management Toronto Metropolitan University Toronto, ON Canada; 3 Division of Geriatric Medicine Department of Medicine University of Toronto Toronto, ON Canada

**Keywords:** telemedicine, virtual care visit, geriatric care professionals, aging population, Consolidated Framework for Implementation Research, geriatric care, older adults, elderly care, telehealth, digital health, COVID-19, pandemic, technology usability

## Abstract

**Background:**

Geriatric care professionals were forced to rapidly adopt the use of telemedicine technologies to ensure the continuity of care for their older patients in response to the COVID-19 pandemic. However, there is little current literature that describes how telemedicine technologies can best be used to meet the needs of geriatric care professionals in providing care to frail older patients, their caregivers, and their families.

**Objective:**

This study aims to identify the benefits and challenges geriatric care professionals face when using telemedicine technologies with frail older patients, their caregivers, and their families and how to maximize the benefits of this method of providing care.

**Methods:**

This was a mixed methods study that recruited geriatric care professionals to complete an online survey regarding their personal demographics and experiences with using telemedicine technologies and participate in a semistructured interview. Interview responses were analyzed using the Consolidated Framework for Implementation Research (CFIR).

**Results:**

Quantitative and qualitative data were obtained from 30 practicing geriatric care professionals (22, 73%, geriatricians, 5, 17%, geriatric psychiatrists, and 3, 10%, geriatric nurse practitioners) recruited from across the Greater Toronto Area. Analysis of interview data identified 5 CFIR contextual barriers (complexity, design quality and packaging, patient needs and resources, readiness for implementation, and culture) and 13 CFIR contextual facilitators (relative advantage, adaptability, tension for change, available resources, access to knowledge, networks and communications, compatibility, knowledge and beliefs, self-efficacy, champions, external agents, executing, and reflecting and evaluating). The CFIR concept of external policy and incentives was found to be a neutral construct.

**Conclusions:**

This is the first known study to use the CFIR to develop a comprehensive narrative to characterize the experiences of Ontario geriatric care professionals using telemedicine technologies in providing care. Overall, telemedicine can significantly enable most of the geriatric care that is traditionally provided in person but is less useful in providing specific aspects of geriatric care to frail older patients, their caregivers, and their families.

## Introduction

Canada’s older population remains at the greatest risk of dying from COVID-19, caused by the novel SARS-COV-2 virus [1]. Yet, the same public health measures being imposed to protect this population have also posed an ongoing challenge for older persons in accessing in-person care in a timely manner since the beginning of the COVID-19 pandemic. In response, the Government of Ontario’s health care system, like several others, rapidly supported the early and widespread adoption and use of its existing and other telemedicine technologies, including the telephone or popular videoconferencing platforms, such as Zoom and Skype, for health care professionals to deliver safe and effective remote or virtual care throughout the COVID-19 pandemic [2].

For older persons, previously noted beneficial outcomes of Ontario’s telemedicine services have included a decrease in wait times for access to specialists [3] and a significant reduction in emergency department (ED) admissions [4]. However, until the beginning of the COVID-19 pandemic, there remained major challenges that hindered the widespread adoption of telemedicine technologies by health care professionals across Ontario. For example, a main barrier was that prior to the COVID-19 pandemic, Ontario physicians could only be reimbursed for providing telemedicine services if they and their patients were able to use the Government of Ontario’s Ontario Telemedicine Network (OTN) secure videoconferencing technologies to conduct patient visits [5]. Telephone-based consultations or follow-up were not reimbursable in general for Ontario physicians, except for geriatricians when providing caregiver advice and support to one of their established patients. Furthermore, another main barrier was that acquiring the required communication technologies to enable secure videoconferencing via telemedicine could be expensive for both patients, their caregivers and families, health care professionals, and their organizations, although this was becoming less of an issue in recent years with the greater availability of secure web-based communication technologies using standard computer equipment. Indeed, many older persons, their caregivers, and their families might also not be able to access the technology needed to use telemedicine services [6]. In addition, many older persons with cognitive and sensory impairments need to rely on health care professionals or unpaid caregivers and family members to assist with or manage the technology [6]. This is a particular concern for those who are homebound or live in isolation, as they may not readily have access to the level of health care professionals or caregiver support necessary for accessing telemedicine technology-enabled supports [7]. Nevertheless, evaluations of Ontario telemedicine programs, prior to the COVID-19 pandemic, have demonstrated high satisfaction among older patient users [3,6,8-11].

Despite positive reported patient outcomes, there remain gaps in the current literature on whether the use of telemedicine technologies can adequately meet the needs of Ontario geriatric care professionals to facilitate the delivery of the range of care they provide. Many observed findings from previous evaluations of Ontario telemedicine programs have reflected the needs of patients and program stakeholders specific to individual conditions, such as telehomecare for older patients with chronic obstructive pulmonary disease (COPD) and heart failure [6], or more specific geographical needs, such as the provision of geriatric medicine and psychiatry outreach clinics to rural and remote communities [9]. Meanwhile, the complexities that geriatric care professionals in Ontario typically manage using a holistic in-person approach that assesses the complex and often interrelated health and social issues experienced by their older patients have not been addressed through prior studies examining the use of telemedicine technologies.

Another issue is that many of the existing Ontario telemedicine programs have tended to target older persons who were more able-bodied, and have often excluded the more vulnerable, older persons with complex conditions [6,12,13]. In particular, health care professionals did not see the benefit of using telemedicine technologies with older persons with physical and cognitive impairments, as they were concerned that this older subgroup of patients could not keep up with the unique demands a remote consultation requires [6,14,15]. However, the exclusion of this subgroup in prior studies has only served to pose a greater challenge for geriatric care professionals in assessing their ability to transition to using telemedicine, particularly with their older patients with complex conditions [16].

At the outset of the COVID-19 pandemic, Ontario geriatric care professionals could not be as selective with their older patients regarding how best to care for them under existing public health measures, including when lockdowns and other restricted visiting orders were enacted and there was a general fear of the possible consequences that could befall older patients with complex conditions seeking nonemergent health care services. Ironically, all these factors could further promote physical and social isolation that increase the risk of worsening functional decline and mental health issues that could actually result in more ED visits and acute hospital admissions [15,17].

Existing telemedicine research also has not addressed how best to facilitate and support the level of care that Ontario geriatric care professionals aim to provide. The current literature indicates that virtual care visits require various ongoing background coordination supports, such as patient data management, patient care monitoring, and facilitation of communications between health care staff involved in care planning [6,15,18]. In addition, telemedicine technologies have often been associated with a limited ability to perform a physical exam [19,20] and a difficulty in observing verbal and nonverbal cues that could impact establishing reliable diagnoses [19,21]. Therefore, the consulting health care professional has often needed to rely on a health care facilitator, whenever possible, who would be on the premises with the patient (eg, a local physician or nurse), as their support, which has been seen as crucial for an effective remote visit [15]. However, in the current COVID-19 pandemic paradigm, consulting health care professionals have also had to serve as facilitators, managing all aspects of the telemedicine visit unless there was a family member, caregiver, or health care professional present to assist with a visit.

Therefore, this study aims to determine the benefits and challenges Ontario geriatric care professionals have faced in using telemedicine technologies to provide routine consultations and follow-up care to their older patients with complex needs, their families, and their caregivers, as well as the conditions under which this method proves to be or not be an effective way to provide care. Moreover, identifying frontline benefits and challenges would also provide new learning opportunities for geriatric care professionals across different health care settings and regions in the use of telemedicine technologies [22], which is still a novel approach in the practice of geriatric medicine [13].

## Methods

### Study Design

This was a mixed methods study that included the following: (1) a survey that inquired about the demographic information of participating geriatric care professionals, their experience, and current satisfaction with the use of telemedicine technologies to provide care to their older patients and their caregivers and (2) a semistructured interview that reflected the objective of the study but also allowed participants to freely express their additional perspectives about the use of telemedicine technologies to provide care. Please see the Multimedia Appendix 1 for the semistructured interview guide.

The Consolidated Framework for Implementation Research (CFIR) was used to assess the barriers and facilitators toward providing care with telemedicine technologies identified in the semistructured interviews. The CFIR was identified as an appropriate methodological framework for providing a comprehensive evaluation of the barriers and facilitators in the implementation of health care technologies across multiple contexts [16,23,24]. The CFIR consists of 5 domains: intervention characteristics, outer setting, inner setting, individual characteristics, and the process of implementation [24]. Within each domain, there are various constructs that guide users to identify barriers and facilitators that impact implementation [24].

### Participants

This study targeted geriatric care professionals who use telemedicine technologies with older patients, their caregivers, and their family members in an outpatient setting to provide routine consultations and follow-up care. The geriatric care professionals were recruited through the Divisions of Geriatric Medicine and Geriatric Psychiatry at the University of Toronto, Canada, and the local Regional Geriatric Program of Toronto between January and April 2021.

A total of 30 geriatric care professionals representing the fields of geriatric medicine, geriatric psychiatry, and geriatric nursing were invited to the survey and participated in a semistructured interview. These geriatric care professionals work in geriatric outpatient clinics that often do not use allied health care workers.

### Ethical Considerations

Participants provided either written informed consent or audio-recorded oral consent. The study protocol was approved by the Toronto Metropolitan University Research Ethics Board (#2020-513-1).

### Data Analysis

The Statistical Package for the Social Sciences (SPSS; IBM Corporation) was used to analyze the survey responses and determine participant characteristics. A deductive thematic approach to analysis was used to analyze the semistructured interviews. Authors WC and AF independently coded the transcripts using the codebook based on the CFIR constructs [24], which was modified to reflect the local geriatric care professionals’ practice. NVivo (March 2020 release; QSR International) was used to facilitate the coding process. Following a codebook helped to minimize coding differences, and weekly discussions were held to resolve coding differences.

## Results

### Participant Characteristics

Of the 30 participants, 22 (73%) were geriatric medicine specialists or geriatricians, 5 (17%) were geriatric psychiatrists, and 3 (10%) were geriatric nurse practitioners. In addition, 28 (93%) participants completed both the survey and the semistructured interview. The survey results of 2 (7%) participants were not collected due to personal choice or technical difficulties. Tables 1 and 2 provide a detailed overview of the participant characteristics and satisfaction with telemedicine use, respectively, from the 28 completed surveys.

**Table 1:** Participant characteristics (N=28).

Participant characteristics		Participants^a^
Age (years), mean (range)		44 (30-74)
**Gender^b^, n (%)**		
	Woman	15 (54)
	Man	12 (43)
**Clinical profession, n (%)**		
	Doctor of medicine (MD)	26 (93)
	Nurse practitioner (NP)^c^	2 (7)
**Medical discipline, n (%)**		
	Geriatrics	23 (82)
	Geriatric psychiatry	5 (18)
**Years of professional experience, n (%)**		
	Less than 3 years	6 (21)
	4-10 years	10 (36)
	More than 10 years	12 (43)
**Patient setting, n (%)**		
	Outpatient	25 (89)
	Other^d^	3 (11)
**Used telemedicine prior to COVID-19, n (%)**		
	Yes	13 (46)
	No	15 (54)
**Experience with telemedicine, n (%)**		
	3-6 months	2 (7)
	6 months-1 year	15 (54)
	More than 1 year	11 (39)
**Frequency of telemedicine use, n (%)**		
	Rarely	1 (4)
	Sometimes	5 (18)
	Often	20 (71)
	Always	2 (7)
**Types of telemedicine platforms, n (%)**		
	OTN^e^ videoconferencing	5 (18)
	Zoom/Skype/Google Hangouts/Facetime	4 (14)
	Combination of telemedicine platforms^f^	19 (68)

^a^The survey results of 2 participants were not collected, 1 participant declined to complete the survey, and 1 participant’s survey was not collected due to technical difficulties.

^b^One participant did not provide gender information.

^c^Both nurse practitioners practiced in a geriatric medicine setting.

^d^The “Other” setting included a combination of an outpatient setting, long-term care homes, and supportive housing.

^e^OTN: Ontario Telemedicine Network.

^f^The combination of telemedicine platforms included Zoom, the OTN, email, telephone WebEx, Facebook Messenger, Microsoft Teams, electronic medical record (EMR)-based applications, WhatsApp, and Facetime.

**Table 2:** Geriatric care professional telemedicine satisfaction survey (N=28).

Questions	Strongly disagree, n (%)	Disagree, n (%)	Neutral, n (%)	Agree, n (%)	Strongly agree, n (%)
1. Telemedicine can increase my productivity in delivering patient care.	0	4 (14)	5 (18)	10 (36)	9 (32)
2. My patients provide me with sufficient information about their comorbidities using telemedicine.	1 (4)	3 (11)	4 (14)	16 (57)	4 (14)
3. I can conduct a comprehensive geriatric assessment using telemedicine^a^.	0	8 (29)	1 (4)	16 (57)	3 (11)
4. Telemedicine services do not require a lot of training to use^a^.	1 (4)	10 (36)	4 (14)	12 (43)	1 (4)
5. Telemedicine services are compatible with the existing clinical workflow.	1 (4)	4 (14)	2 (7)	17 (61)	4 (14)
6. Teleconsultation is as effective as an in-person consultation^a^.	3 (11)	12 (43)	5 (18)	8 (29)	0
7. My older patients can easily communicate with me using telemedicine.	2 (7)	10 (36)	10 (36)	5 (21)	0
8. I can engage with my patients, their families, and their caregivers about treatment plans using telemedicine.	0	1 (4)	5 (18)	18 (64)	4 (14)
9. I would continue to use telemedicine to care for my older patients beyond the pandemic.	0	2 (7)	2 (7)	14 (50)	9 (36)
10. Overall, I am satisfied with using telemedicine with older patients.	0	2 (7)	6 (21)	17 (61)	3 (11)

^a^The percentages do not add up to 100% due to rounding.

### Barriers to and Facilitators of Telemedicine Use

Table 3 details the barriers and facilitators associated with the implementation of telemedicine in geriatric care practice. Only relevant key constructs identified within the 5 CFIR domains are discussed herein.

**Table 3:** Adapted CFIR^a^ operational codes.

Domains and constructs		Operational definition^b^	Facilitator/barrier
**I. Intervention characteristics**			
	Relative advantage	Perception of geriatric care professionals seeing virtual care visits as an advantage versus in-person consultations	Facilitator
	Adaptability	The degree to which the virtual care visit was tailored to meet the needs of geriatric care professionals	Facilitator
	Complexity	Perceived complexity of how virtual care assessments compared to in-person assessments	Barrier
	Design quality and packaging	Perceived quality of telemedicine platforms and how the innovation is bundled and presented	Barrier
**II. Outer setting**			
	External policy and incentives	Broad constructs on government policies, such as confidentiality issues/consent with older patients, as well as discussions about how to bill for virtual care visits (consults via telephone, text messages, or videoconferencing)	Neutral
	Patient needs and resources	The degree to which the needs of older patients with complex needs, their caregivers, and their families are accurately known and prioritized during virtual care visits	Barrier
**III. Inner setting**			
	Networks and communications	The quality of information derived from fellow colleagues, caregivers, families, and local EMR^c^ systems to develop collateral history regarding older patients with complex needs	Facilitator
	Culture	Norms, values, and basic assumptions of geriatric care professionals toward telemedicine use prior to the COVID-19 pandemic	Barrier
	Implementation climate: tension for change	The degree of willingness to transition to telemedicine use	Facilitator
	Implementation climate: compatibility	The degree of tangible fit between meaning and values attached to virtual care visits, how those align with the geriatric care professionals’ own norms, values, and perceived risks and needs, and how virtual care visits fit into the existing workflow and systems	Facilitator
	Readiness for implementation	Geriatric care professionals’ readiness to implement virtual care visits	Barrier
	Readiness for implementation: access to knowledge and information	Ease of access to training and support provided on how to conduct virtual visits	Facilitator
	Readiness for implementation: available resources	The level of resources provided for telemedicine use, including technological infrastructure, dedicated clinic space to conduct virtual care visits, and educational guidance	Facilitator
**IV. Individual characteristics**			
	Knowledge and beliefs about the intervention	Geriatric care professionals' attitudes toward the values placed on virtual care, as well as familiarity with facts, truths, and principles related to telemedicine technologies	Facilitator
	Self-efficacy	Geriatric care professionals' beliefs in their own capabilities in using telemedicine technologies with older patients, their caregivers, and their families	Facilitator
**V. Implementation process **			
	Engaging: champions	Individuals who drove the implementation of virtual care visits forward	Facilitator
	Engaging: external change agents	Outside individuals who formally influenced or facilitated virtual care visit decisions in a desirable direction	Facilitator
	Executing	Carrying out and accomplishing tasks during care visits	Facilitator
	Reflecting and evaluating	Quantitative and qualitative feedback on progress and quality to enhance virtual care visits	Facilitator

^a^CFIR: Consolidated Framework for Implementation Research.

^b^The operational definitions of the constructs are adapted to reflect the geriatric care professionals’ experiences.

^c^EMR: electronic medical record.

### Domain I: Intervention Characteristics

#### Relative Advantage

Using telemedicine technologies increased access to older patients who were homebound, were reluctant to come in due to COVID-19, or lived in remote areas. Many geriatric care professionals perceived that virtual care visits allowed older patients the convenience to receive care in their own homes without the hassle of traveling. Older patients who were previously “no shows” for their appointments were also more likely to be reached. Virtual care visits also provided greater schedule flexibility for geriatric care professionals to accommodate the schedules of their older patients, their caregivers, and their families more easily.

I started with this idea that there are certain patients, maybe like homebound people, who would be very difficult for them to come into clinic. And so, these kinds of people, I can provide service to who I wouldn't have been able to otherwise. Patients who are [reticent] to be in person because of the pandemic would feel comfortable that way. You know…there are some people who I would not have been able to evaluate if it were not for virtual meet means.

Geriatric care professional 1

I think one thing is because it works so well for their schedule, for their lives. Now that they can call, they can maybe work in the morning, and then they have a break from like, say, 10 to 11…So, we've had a lot more people just working the same day, but they had a break, or they took an hour off work, and they were able to just do the Zoom or the virtual, and then it worked out quite well. So yeah, I think for families and for caregivers, it was definitely a convenience.

Geriatric care professional 26

#### Adaptability

Many statements revealed that geriatric care professionals found ways to adapt their assessments virtually. Adapted methods included having the family members or caregivers assist patients in conducting tests, developing different backup communication plans, using modified clinical assessment tools, or collecting more information that was presently available.

Often what I was doing with the family members’ assistance was just asking them to test strength. So, asking the [patient to] put their hands up, and then having the family member just press down and tell me is there resistance there, or do they just collapse? And oftentimes, I can see that over the video if they just collapse. So those are kind of the things that we would collect over video.

Geriatric care professional 15

I surprised myself that you can actually do geriatric psychiatry for the most part, on a video, and/or a telephone, which is about half my patients who do not have a computer. And maybe another 10% who don't know how to use it when they own it, and so, I conduct at least half of my interviews by telephone rather than video.

Geriatric psychiatrist 17

And then when we come to the physical exam, then…I would mute the OTN, so there's no feedback, and I would talk to [the patient] on the phone and watch them on the video. So, there's a lot of creativity that needs to happen in order to make these things go smoothly when, and not all the elderly people have a younger person, like a family member, who can physically go and help them get online.

Geriatric care professional 20

The cognitive piece, we have several tools, which can be administered virtually, like [the] MoCA [Montreal Cognitive Assessment], sharing the screen on Zoom, and you can guide people through the virtual exam. Even on the telephone, there are there are ways of doing certain [parts] of the mental status exams…you can certainly assess for depression because you ask people questions about that.

Geriatric care professional 13

So, what I've been doing is even more detailed functional history, particularly focusing on what can you do, but how has it changed over the last 6 months and things like that to see if there's a progressive nature, which I think is sort of a…you know, that's what we're worried about with cognitive decline is that there's going to be functional decline. So, I have substituted in the virtual platform [a] more detailed, functional history instead of doing [a] detailed cognitive history.

Geriatric care professional 5

#### Complexity

The complexities of using telemedicine technologies with older patients, their caregivers, and their families was an evident barrier. Geriatric care professionals discussed the following challenges: (1) navigating the transition from in-person consultations to virtual care; (2) establishing interpersonal connections with their patients for new consults; (3) difficulty conducting comprehensive geriatric assessments (CGAs), especially the physical exam component; (4) engaging with older patients with sensory or cognitive impairments or behavioral issues; and (5) gathering sufficient collateral information from caregivers, families, or information systems.

So initially, our nurses and even our admin, in terms of booking, there have been concerns about, well, who are we supposed to be emailing? Who are we calling? And trying to make sure we have all the right players in place because we can't see Power of Attorney documents. We need to be making decisions regarding the [institutional] hierarchy. We don't know all of that stuff up front.

Geriatric care professional 1

Part of medicine is the patient interaction, the physical exam, seeing how the person walks and moves and talks. That is one element that is missing is you cannot examine the patient beyond the very, very basic exam. Even the cognitive paper exams are limited. So, all of that needs to be taken very much into account. Certain assessments are just not going to be possible...are not going to be as accurate because of that absence.

Geriatric care professional 4

The problem of course is not all doctors’ files are available there. And none of the family doctors that I know of have any of their notes available on Connecting Ontario. So, you miss that real background importance that family doctors have.

Geriatric care professional 5

The other kind of major area or barrier is that sensory impairments like hearing impairment or visual impairments certainly were barriers. And patients with cognitive impairment…it’s already, like, it's an unfamiliar person, potentially, especially for a new consult. And it's a sort of disembodied head, like over the screen, and they sort of don't necessarily know what's going on. And so that was a little bit more difficult to establish rapport.

Geriatric care professional 8

Again, if patients are very cognitively impaired, they tend not to understand what's happening. I had one lady who, like, was literally running away from the staff member with the camera because she was quite paranoid, and she thought she was being filmed and she was kind of covering her face.

Geriatric psychiatrist 25

The biggest challenge is actually building a human connection with the patient. So, that's been very difficult; especially we have a lot of new consults, and we're trying to bring as few people into the hospital as possible. So, they hear my voice. They will sometimes see me on video, and then same with our nurse practitioners and our occupational therapist in [the] clinic. But, it's not the same. So, they don't get that same kind of connection. You don't build those same bonds.

Geriatric care professional 19

#### Design Quality and Packaging

Geriatric care professionals used a variety of videoconferencing platforms and the telephone to conduct virtual care. However, many statements revealed that connectivity issues were often still a barrier for both geriatric care professionals and their older patients in using videoconferencing platforms.

Regarding the types of telemedicine technologies, many statements indicated a preference for videoconferencing over telephone communications as the geriatric care professionals could see their older patients and their living environments. Many statements also revealed that geriatric care professionals would like videoconferencing platforms to have the ability to facilitate more interactions with their older patients. However, some indicated their older patients preferred telephone communications. In addition, some found that their older patients with hearing impairments could hear better since there was the ability to adjust volume. Few geriatric care professionals indicated they used email communications or text messages with their older patients.

So, I think the connectivity has been a major issue. So oftentimes, you'll lose audio or things are so delayed, that it's really a barrier to communicating with the individual on the other side.

Geriatric care professional 1

Like it would be actually, really nice to have some sort of digital interface where I could write something on the screen, and they could like circle it, or I can show them the cognitive testing on the screen, and they could like draw it on the screen. So, there's a bit more interaction.

Geriatric care professional 15

The only thing we can do with them is telephone, okay with this, which is quite suboptimal because you can't actually see how they're doing. And they will just tell you whatever. And the patients don't know to report certain symptoms that may be concerning on the telephone, whereas, like, if you have a visual of the patient, you're more likely to not miss something, for example.

Geriatric care professional 20

So, to meet somebody new and to be spilling out your guts when you don't see the person, I think it is very hard. I think that is why in those cases, the video is really, really important.

Geriatric psychiatrist 17

### Domain II: Outer Setting

#### External Policy and Incentives

Confidentiality and the security of networks were not a major concern for geriatric care professionals. Several geriatric care professionals questioned the future payment model and discussed the need for more guidance on the billing process for virtual visits.

So, there is a statement that we use from OMA [Ontario Medical Association]. So, we read that out to [the patient], or I usually send it to [the patient] by email. And I then document that in my consult note that your consent was reviewed and accepted.

Geriatric care professional 7

And unless the government is prepared to pay a hell of a lot more for the geriatricians’ time, or, and can continue with these billing codes that they have suddenly sprung up.

Geriatric care professional 13

I do think that if you're going to have a certain service, and meet a certain standard, then be reimbursed at a certain level because there are medical legal implications as well as providing appropriate service. We have to be very clear on what we can and cannot provide, and what we should [and] should not be reimbursed for.

Geriatric care professional 28

#### Patient Needs and Resources

Many statements revealed that telemedicine visits are more effective if the patient has their own monitoring devices that could provide clinical health information, such as vital signs, and a caregiver, family member, or health professional to assist the patient during the visit. However, many expressed concerns about the support and available resources for their older patients to use telemedicine. Several also expressed concerns about the health and technology literacy of their older patients and caregivers.

So, blood pressure, usually I can get by because most families will have a blood pressure machine at home, which I recommend they bring with them so that they're able to do that for me virtually.

Geriatric care professional 3

I think a huge portion of the people I saw had caregivers who were helping navigate this, and, and the few that didn't, like some of them were able to, but those were the less impaired. The more impaired who weren't able to, if we didn't have nurses who were able to go there to support them to get onto the video call, there'd be no way.

Geriatric care professional 15

The big thing is that you are missing a lot of people that you don't even know, like all those who don't have technology, or all the people that may live in public housing, that are poor…those are individuals who probably would have come to the hospital, but who don't have the web; who don't have the iPhones, or iPads or, or, high technology, and we're missing them.

Geriatric care professional 13

People who are more health literate, and more technology literate, and have reasonable education, of course, those are moderately correlated with each other, are probably the best ones to be able to do the televisits with ideally…if they don't have a caregiver and they live alone and they are cognitively impaired or they don't speak English fluently…they are [the] ones that I think would be less well served by telemedicine.

Geriatric care professional 28

### Domain III: Inner Setting

#### Networks and Communications

Many statements revealed geriatric care professionals relied on quality collateral information (eg, patient medical history) derived from their older patients’ caregivers, families, referring physicians, local team members, and electronic medical record (EMR) systems.

I think that in the nature of how geriatrics sort of works in general, you can get a lot of information just from, sort of, descriptive, you know, scenarios and, sort of, gaining that information, enough to make significant changes and significant improvements. And even though it would not be perfect, I find that that there is still a lot of good work that can be done.

Geriatric care professional 10

Then, you mentioned the medication compliance. So that's where collateral is really important. So, we really rely on family and caregivers to tell us that, you know, the blister pack…they're pretty reliable with it…or I came by the other day, and there's three days of missing meds. So, we can't physically see the blister pack but except for video. Actually, if they have video, sometimes we do have them show it to us. But usually, the collateral there can help verify that, I think, almost just as well as if we were there in person.

Geriatric care professional 19

#### Culture

Prior to the COVID-19 pandemic, using telemedicine technologies was not the norm for geriatric care professionals. Geriatric care professionals preferred to be on-site with their patients and fellow colleagues.

I had historically rejected participating in the Ontario Telemedicine Network. I just wasn't interested. It was a variation on home visits from my viewpoint. And I prefer to sit and do what I did, which was see the person with their family, have that direct interaction, and proceed from there.

Geriatric care professional 11

#### Implementation Climate

This construct was broken into two subconstructs: tension for change and compatibility.

##### Tension for Change

The rapid implementation of telemedicine technologies by geriatric care professionals across Ontario was due to the COVID-19 pandemic.

Now when COVID hit, I had no choice, and we all went virtually.

Geriatric care professional 10

##### Compatibility

Many geriatric care professionals perceived that telemedicine technologies are highly compatible for addressing polypharmacy issues, effectively conducting follow-up care, and inquiring about patient medical histories. Geriatric psychiatrists found telemedicine technologies to be compatible with their clinical practice.

And so often, I think it's good for maybe follow-ups where, especially if it's a complex case, good to see the person in person. But then, if you just want to follow up and see how the pain is, see how they're doing cognitively, then you can do that very comfortably virtually.

Geriatric care professional 4

So, one of the big issues that older adults often face is polypharmacy, and with, like, video chatting, audio chatting, we were still able to review their medications, review the indications, side effects, what issues they were having, whether it was timing, being in bottles, it's not blister-packed. That was a big piece of a lot of the assessments or is still a big piece of a lot of the assessments. So, that was very, very helpful to still be able to do that part of the assessment.

Geriatric care professional 15

And I think it might be very different if you're geriatric physician, who [deals] with a physical problem, as opposed to a psychiatrist, where most of the [problems] we deal with are mental or psychological and, therefore, can be assessed by questions.

Geriatric psychiatrist 14

#### Readiness for Implementation

Many geriatric care professionals found the transition to virtual care visits was unexpected and sudden. However, a few statements also revealed that those who had previous experience with telemedicine use found the transition to be smooth.

I've already started using that in my training at [Medical Institution] before transitioning into practice. So, it wasn't a big transition for me, and I find it pretty easy to use.

Geriatric care professional 3

The training and my comfort in terms of making assessments in person that had been honed, both consciously and subconsciously, over 20 years, [have] now been abruptly changed to filter through a screen.

Geriatric care professional 12

This construct was also broken down into two subconstructs: available resources and access to knowledge.

##### Access to Knowledge

Several geriatric care professionals indicated they could adopt the training on telemedicine into their clinical practice, but there was still a learning curve.

Most of the session was talking about how challenging it is, which we all knew, it was very challenging…Some of the cognitive assessment ideas we got from that part of the workshop and incorporated them and just kind of adopted it from there.

Geriatric care professional 6

I think there were opportunities by OMA. There were webinars. And so yes, if somebody really needed to learn it or had questions, I think there were opportunities available for them. But of course, you had to do your work. There was a learning curve. You need to get used to it.

Geriatric care professional 7

##### Available Resources

Many geriatric care professionals had available resources and support for using communication infrastructure, standardized clinical assessment tools, and training on virtual care. A few did not initially have infrastructure available to them.

We did have the appropriate support in our hospital for OTN connections. We did have appropriate support in the hospital to provide the technical support to be able to do all of this, telemedicine from home actually, from my office, which currently is at home. And this was very helpful.

Geriatric care professional 2

### Domain IV: Individual Characteristics

#### Knowledge and Beliefs About the Intervention

Many geriatric care professionals perceived that virtual care visits will be incorporated into their clinical practice in the future due to their benefits in reaching their older patients.

I think that there's definitely some of the benefits that I think are helpful…is that we have more options now. I think that it'll sort of carry over. I think, post–COVID, of having sort of the options to have different pathways to see our patients. If for whatever reason people can't come in, then our options were home-visiting teams, or things like that, that may have [been much] more limited in the sense of [a] longer waitlist. So, I think that that benefited that accessibility.

Geriatric care professional 10

#### Self-Efficacy

Many geriatric care professionals were confident in using telemedicine technologies to meet the care needs of their older patients with complex conditions, their caregivers, and their families. However, some were still apprehensive about their ability to conduct care virtually.

But like, I'd say, like 90% of the encounters, I was pretty satisfied with that I had achieved kind of the same level of assessment that I would have otherwise.[Geriatric care professional 8]

And so aside from accuracy of diagnosis, I wonder if my therapeutic presence, which can be hard to quantify, is lost over a virtual platform? Or does the individual feel the same degree of therapeutic presence with an office virtual assessment?

Geriatric care professional 12

### Domain V: Implementation Process

#### Engaging

This construct was broken down into two subconstructs: champions and external change agents.

##### Champions and External Change Agents

Several statements indicated that having a champion in the team or an external role model helped facilitate the implementation of telemedicine technologies.

We, as a clinic, were very lucky to have a clinician, which was just on top of all of these new changes, and [were] able to switch from seeing patients in person to telemedicine.

Geriatric care professional 2

#### Executing

Several geriatric care professionals found that they were more efficient with time during the virtual care visit, but it did not increase their patient capacity. Some statements revealed that there was additional follow-up work required with telemedicine use, especially if the older patient needed to be followed up in person.

When you're in [an in-person] clinic situation, the nurse or someone is going pop their head in and say, “[Doctor], the next patient is waiting.” In a virtual, I've no one managing my time aside from me. So, I am much more efficient. If I have a 30-minute telephone assessment, I'm out at 30…because historically, I would gauge my time, and it was just about it.

Geriatric care professional 11

But, I guess the biggest increase to workload is that if I determine that we need to, I need to, see them, then it's another visit soon afterwards. And oftentimes, it's a home visit. And yeah, that adds time.

Geriatric care professional 6

#### Reflecting and Evaluating

Several statements revealed that geriatric care professionals would frequently reconvene with their clinical teams or peers to evaluate their experiences with telemedicine use.

And we would also meet with the RGP, which is the Regional Geriatric Program. And it was weekly meetings to kind of discuss what's working, what's not working. How are you guys using your referral forms? How are you generating email addresses? So, it was a lot of communication within the city, interestingly enough, to actually get these programs up and going. And it was cool because it was great to get those perspectives from interdisciplinary teams. I think that was the most important piece, that it was all members, so physician, administrative assistant, nurse practitioner, OT, PT, everybody was feeling it. So, we all had to sort of pitch in and collaborate.

Geriatric care professional 23

## Discussion

### Principal Findings

In this study, the CFIR was used to develop a comprehensive narrative of the current experiences of geriatric care professionals in routinely using telemedicine technologies in Ontario in the light of the COVID-19 pandemic. Although 5 barriers and 1 neutral construct were identified, so too were 13 facilitators.

This mixed methods study adds to the growing literature on the use of telemedicine to provide geriatric care before and during the COVID-19 pandemic, and we found similar findings to other recent studies that also explored geriatric care professional experiences with the use of telemedicine during the COVID-19 pandemic [22,25-29]. The ubiquitous transition to using telemedicine in the provision of geriatric care was uniformly driven by the COVID-19 pandemic. Geriatric care professionals faced an initial learning curve as they learned to incorporate telemedicine technologies into their routine clinical practices. However, for geriatric psychiatrists specifically, there was a more seamless transition to virtual care due to the nature of their clinical practice that has always been more amenable to the use of telemedicine technologies, which differs from geriatricians and geriatric nurse practitioners. For example, the physical examination is usually not necessary to complete a psychiatric assessment [30,31]. Meanwhile, the main challenge for geriatric psychiatrists was using telemedicine technologies with older patients with severe cognitive impairment. In a systematic review on telemedicine and dementia, Sekhon et al [15] had identified that in-person consultations are more appropriate for this subset of older patients with complex conditions.

Our study findings also raised additional unique insights being experienced by Ontario geriatric care professionals. Notably, our study was able to explore the range of strategies adopted by Ontario geriatric care professionals to complete their clinical assessments virtually, whereas other recent studies have largely focused on navigating the technological aspect of telemedicine use to overcome barriers [22,26]. As noted in *Adaptability*, Ontario geriatric care professionals quickly adopted the use of validated clinical tools that could enable them to virtually conduct their assessments or better prioritize assessment components when certain aspects were hindered by the challenges in using telemedicine technologies. Thus, with regard to improving telemedicine technologies to facilitate the more effective provision of geriatric care, Ontario geriatric care professionals would like videoconferencing platforms to have the ability to facilitate more interactions with their older patients, such as the capability to see how their older patients complete the actual written exercises in the validated clinical tools they use. Observing how their older patients actually complete these exercises, such as drawing a picture or connecting dots, provides important insights for the geriatric care professionals regarding the physical and cognitive abilities of their older patients. Another notable finding to support overcoming identified barriers was around the role of collateral information derived from caregivers, friends, family members, referring colleagues, and EMR systems. The responses of our study participants illuminated the importance of collateral information, as discussed in the *Networks and Communications* construct which played a crucial role across the whole implementation process in the virtual delivery of care. However, gathering sufficient collateral information was a complexity for our study participants, while Watt et al [28], in a recent study, had found that the persistent need to collect collateral information is a complexity for virtual care. Nevertheless, collateral information helps provide a comprehensive overview of the patient’s medical and social history for geriatric care professionals without needing to see the patient in person to derive this. Furthermore, linkable EMR data were associated with more opportunities for understanding the patient journey through the care continuum [32]. Hence, geriatric care professionals were often still able to make effective clinical decisions virtually for their older patients when given sufficient collateral information that helped compensate for the other factors that can limit the usefulness of telemedicine. Watt et al [28] had also indicated that geriatric care professionals found collateral history to be particularly useful for telephone assessments in which visual assessment was not possible.

Due to the inherent challenges that exist in using telemedicine technologies, the *Compatibility* construct revealed a consensus from the responses of study participants that the role of telemedicine technology for Ontario geriatric care professionals was more appropriate for follow-up visits. Participants reasoned that follow-up visits do not require as comprehensive assessments as an initial consultation that would likely have components that benefit from an in-person assessment, such as a physical examination or certain cognitive tests. This aligns with findings by Watt et al [28] and studies that have evaluated physicians’ experiences in telehealth visits with older patients in the context of US practice [26,29].

Regarding the observed perceptions of patient needs and resources, the responses of this study’s participants echoed concerns around the “digital divide,” which continues to be a major barrier for older persons to using telemedicine technologies in Ontario [8,25,26,33]. In particular, their responses revealed important necessary aspects for an effective virtual care visit to take place without the assistance of a health care facilitator present with their older patients. Many of our study participants had indicated that their older patients often relied on the presence of a caregiver or a family member to access the communication technology and to manage the virtual care visit. This is also supported in recent studies in which primary care physicians found the assistance of family members and caregivers to be helpful in the facilitation of the telehealth visit for their older patients [22,29]. Additionally, our study found that the visits were even more effective for older patients who had monitoring devices that could provide basic health information, but not all older patients owned these devices. However, the varied level of health and technology literacy of their older patients and their caregivers or family members posed challenges for the ability of geriatric care professionals to collect information for their clinical assessments virtually.

The responses of our study participants suggest that there exist three necessary aspects to achieve an effective virtual care visit for both geriatric care professionals and their older patients: (1) access to the telemedicine visit–enabling technology (smartphones, tablets, computers, or telephone); (2) access to health-monitoring equipment to provide basic health information, such as blood pressure monitors; and (3) appropriate health and technology literacy amongst older patients and their caregivers or family members. Essentially, older patients or their caregiver or family member would need to assume the traditional role of the on-site health care facilitator. The reality, however, is that only a certain portion of older Ontarians have the means and ability to support the effective use of telemedicine services. It is less likely for older persons to use telemedicine technologies if they lack confidence with using related technologies [11,22,26] or to receive virtual care visits via videoconferencing if their caregivers could not be present [34]. Recent studies also indicate that older persons at the lower end of the socioeconomic spectrum are often overlooked as they lack equitable access to the appropriate resources and support to facilitate virtual care visits [25,26,35], and geriatric care professionals have observed worsening of this disparity during the COVID-19 pandemic [25]. This is important to note since a main purpose of telemedicine technologies is to bridge the gap in care accessibility for older persons who live in low-resource settings [36].

Lastly, an important consideration is how evolving policies and incentives could fundamentally change the landscape for providing virtual care visits in Ontario. As discussed in the External Policy and Incentives section, our study’s geriatric care professionals raised concerns, including around the ambiguity about future billing processes for the provision of virtual care visits. Although the Government of Ontario quickly implemented temporary billing codes and guidelines to facilitate the provision of virtual care visits at the start of the COVID-19 pandemic [37], the future funding model for virtual care visits in Ontario will largely reflect the recent impact on the use of telemedicine technologies due to the COVID-19 pandemic [2,26]. If Ontario geriatric care professionals continue to embrace the use of virtual care visits, future funding policies will need to determine how to broadly support the appropriate use of telemedicine to provide high-quality geriatric care, while recognizing there still exist socioeconomic barriers to accessing it and trade-offs related to its use [28].

### Strengths and Limitations

This is the largest known study pertinent to the real-world experience of geriatric care professionals using a wide range of telemedicine technologies in the light of and during the first year and a half of the COVID-19 pandemic. This study further used the CFIR to provide a comprehensive overview of the various strategies geriatric care professionals have used to overcome the complexities surrounding the provision of outpatient virtual care with older persons, their caregivers, and their families. Another strength is that this study included a wide-ranging age group of geriatric care professionals. In addition, this study primarily focused on the experiences of geriatric care professionals and did not evaluate older patients’ and their caregivers’ perspectives. Yet, despite the lack of these perspectives, responses across all the constructs were effective in revealing the various practice changes and strategies used to address the diverse needs of older patients with complex conditions in the virtual care setting.

There are several limitations to the study. First, the findings are limited to the experiences of geriatric care professionals in Canada’s Greater Toronto Area. In addition, the experience of nonmedical or nursing allied health professionals, who are also integral members of some geriatric care teams, was not included. Nevertheless, the majority of Ontario’s geriatric care professionals work in the Greater Toronto Area, with the vast majority being geriatricians, geriatric psychiatrists, and nursing care professionals who were included in this study. In addition, this study’s CFIR construct *Reflecting and Evaluating* indicated that geriatric care professionals are continuously evaluating their own experiences that shape their knowledge and beliefs about the use of telemedicine in their practices. Hence, the findings presented herein should represent a snapshot of the current needs of geriatric care professionals that will likely evolve as we continue to navigate the opportunities to using telemedicine technologies to deliver geriatric care.

### Recommendations

Based on the findings of this study, we offer the following four recommendations to support the continued and enhanced use of telemedicine technologies by geriatric care professionals in providing care to older patients, their caregivers, and their families:

1. Continuing training and education for geriatric care professionals in the use of telemedicine technologies is needed: Prior training on telemedicine use had helped facilitate a smoother transition for geriatric care professionals during the rapid transition to predominantly providing virtual care visits at the outset of the COVID-19 pandemic. In addition, the use of telehealth interventions relies on the experiences of clinicians in using the technology as intended [16]. Hence, continuing education can provide new learning opportunities for the best use of telemedicine technologies for geriatric care professionals [22].
2. Training in the use of telemedicine technologies is needed for older patients, their caregivers, and their families, as well as on how to collect basic health information that may facilitate a telemedicine assessment. This could help alleviate some of the challenges in obtaining clinical information and further enhance the feasibility of virtual care visits without the presence of a health care facilitator with the patient. As McLean et al [11] noted, providing basic training for older patients, their caregivers, and their families could help them better navigate and feel more comfortable in using various telemedicine technologies.
3. Health care systems should maintain virtual care visits as an option available to older patients, their caregivers, and their families, with geriatric care professionals when this option may represent an equally or better way to facilitate care. This recognizes that virtual care visits give older patients, their caregivers, and their families and geriatric care professionals more flexibility to both provide and receive care. It should represent a mechanism through which to provide older patients, their families, and caregivers with the appropriate community infrastructure supports that could help reduce barriers for older patients, their caregivers, and their families in accessing telemedicine technologies.
4. Ensure that future reimbursement models to enable telemedicine or virtual care visits are financially sustainable for geriatric care professionals. Virtual care visits will likely be incorporated into the future provision of geriatric care in Ontario. Hence, temporary payment models will likely transition into long-term ones, and policy makers will need to ensure that the long-term methods of funding the provision of telemedicine-based care are financially sustainable, while ensuring the needs of geriatric care professionals and older persons can also be met.

### Conclusion

The sudden need to find alternative ways to provide care in safe and effective ways at the outset of the COVID-19 pandemic forced health care systems worldwide to enable the rapid and widespread adoption and use of telemedicine technologies by geriatric care and other health care professionals. Overall, this study found that Ontario geriatric care professionals could adapt the use of telemedicine technologies to provide virtual care to meet the complex needs of their older patients, but there also exists a threshold in their ability to effectively provide geriatric care using telemedicine technologies. Indeed, geriatric care professionals have been found to perceive telemedicine technologies or virtual care methods to be more appropriate in the provision of follow-up visits that do not usually require specific assessments that are better done in-person. However, this study also noted that there are also various additional issues that will prohibit the greater widespread and permanent adoption of telemedicine technologies in Ontario, especially in the provision of geriatric care, unless specifically addressed. Further research on addressing older patient equity and inclusion, medical information infrastructure, and economic policies will be beneficial for understanding the best practices for supporting the use of telemedicine technologies to provide both more effective and equitable geriatric care.

## Appendix

Multimedia Appendix 1 Geriatric Care Professional Semistructured Interview Guide.

## Abbreviations

CGA: Comprehensive Geriatric Assessment
CFIR: Consolidated Framework for Implementation Research
COPD: Chronic obstructive pulmonary disease
ED: Emergency department
EMR: Electronic medical record
MoCA: Montreal Cognitive Assessment
NP: Nurse Practitioner
OT: Occupational Health Therapist
OMA: Ontario Medical Association
OTN: Ontario Telemedicine Network
PT: Physical Therapist
SPSS: Statistical Package for the Social Sciences
REB: Research Ethics Board
RGP: Regional Geriatrics Program
