# Supplementary material for: Understanding the Experience of Geriatric Care Professionals in Using Telemedicine to Care for Older Patients in Response to the COVID-19 Pandemic: Mixed Methods Study
Source: JMIR Aging. 2022 Aug 10;5(3):e34952. doi: 10.2196/34952 (PMC9369613; doi:10.2196/34952)
Supplement: Multimedia Appendix 1 [file aging_v5i3e34952_app1.pdf]

TED ROGERS SCHOOL
Health Services Management

Semi-structured Interview Guide

- **1). How has your transition to delivering care via telemedicine been?**

Probe the following:

- Were you using any telemedicine methods to see your patients before the pandemic? For example, telephone or video consultations or follow-ups?

- Would you say you are now confident in using telemedicine with your older patients?

- Were there any resources made available to help you integrate telemedicine into your practice?
    - Did you receive any training/guidance/support? What was most helpful/useful?

2). What are the benefits and challenges you face as a provider using telemedicine with your older patients with complex needs, their families and caregivers?

Probe the following:

- How does it compare to conducting a traditional outpatient consult/in-person geriatric assessment or follow-up?

- How do you use telemedicine to assess your patients' ongoing issues like high blood pressure, heart failure, frailty, or dementia?

    - How do you go about certain components of the assessment, if you can't conduct them using telemedicine?

    - What types or other sources of clinical information can you also rely on from referring or involved colleagues?

- How do you address confidentiality with your patients, their caregivers and families? Are you documenting consent?
  Did you receive any training/guidance/support? What was most helpful/useful?

3). Do you think telemedicine services are useful for conducting comprehensive geriatric assessments or follow-ups?

Probe the following:

- Do you think telemedicine can help you deliver care effectively?

- Would you say your patients, their families and caregivers are just as engaged in their treatment plan using a telemedicine platform to deliver care?

4). Do you think telemedicine services are appropriate for managing the care of older patients with complex needs, their families and caregivers?

Probe the following:

- How do you use telemedicine to assess your patients' ongoing issues like high blood pressure, heart failure, frailty, polypharmacy, falls or dementia?
    - How do you go about certain components of the assessment, if you can't conduct them using telemedicine?

- When do you look towards using an alternative or more suitable method to deliver care?

4). Wrap-Up Questions

- Did you incur any costs using telemedicine with your patients?
- If there are plans to continue or expand geriatric telemedicine services, what areas would need improvement? (For example, financial subsidies, better guidance/framework, specific resources…etc..)
